# The living microarray: a high-throughput platform for measuring transcription dynamics in single cells

**DOI:** 10.1186/1471-2164-12-115

**Published:** 2011-02-16

**Authors:** Saravanan Rajan, Haig Djambazian, Huan Chu Pham Dang, Rob Sladek, Thomas J Hudson

**Affiliations:** 1 Department of Human Genetics, McGill University, Montréal, Québec, Canada; 2 Department of Medicine, McGill University, Montréal, Québec, Canada; 3 McGill University and Genome Quebec Innovation Centre, Montréal, Québec, Canada; 4 Department of Molecular Genetics, University of Toronto, Toronto, Ontario, Canada; 5 Department of Medical Biophysics, University of Toronto, Toronto, Ontario, Canada; 6 Ontario Institute for Cancer Research, Toronto, Ontario, Canada

## Abstract

**Background:**

Current methods of measuring transcription in high-throughput have led to significant improvements in our knowledge of transcriptional regulation and Systems Biology. However, endpoint measurements obtained from methods that pool populations of cells are not amenable to studying time-dependent processes that show cell heterogeneity.

**Results:**

Here we describe a high-throughput platform for measuring transcriptional changes in real time in single mammalian cells. By using reverse transfection microarrays we are able to transfect fluorescent reporter plasmids into 600 independent clusters of cells plated on a single microscope slide and image these clusters every 20 minutes. We use a fast-maturing, destabilized and nuclear-localized reporter that is suitable for automated segmentation to accurately measure promoter activity in single cells. We tested this platform with synthetic drug-inducible promoters that showed robust induction over 24 hours. Automated segmentation and tracking of over 11 million cell images during this period revealed that cells display substantial heterogeneity in their responses to the applied treatment, including a large proportion of transfected cells that do not respond at all.

**Conclusions:**

The results from our single-cell analysis suggest that methods that measure average cellular responses, such as DNA microarrays, RT-PCR and chromatin immunoprecipitation, characterize a response skewed by a subset of cells in the population. Our method is scalable and readily adaptable to studying complex systems, including cell proliferation, differentiation and apoptosis.

## Background

A central challenge in the post-genomic era is determining how gene expression is regulated during complex biological processes. Hybridization and sequencing-based technologies such as DNA microarrays and RNA-seq have played a valuable role in identifying and characterizing the components of such processes on a comprehensive scale. Moreover, the combination of these technologies with high-throughput methods for studying protein-DNA and protein-protein binding has enabled us to glean insights into global networks of interactions [1-4]. With the system components coarsely identified, the challenge now lies in the detailed characterization of how transcription of these large sets of genes changes over time and space during normal cellular processes and in response to perturbation.

However, since existing methods of measuring transcription provide discrete measurements of a transcriptional response obtained from large populations of cells, they suffer from two major drawbacks. First, quantifying transcription dynamics using microarrays at multiple time-points is expensive when long processes are under study. Second, despite improvements in assay sensitivity, these approaches typically involve pooling mRNA from thousands of cells. The averaged response measured in this way is adequate for classifying different cell or tissue types, but it is not well-suited for studying processes that show cell-to-cell variation, such as cell division, differentiation, or drug responsiveness. Recent developments in cell-based assays combined with advances in reporter technology allow us to address these limitations, since expression levels can be repeatedly assayed in single cells. Here we describe a method in which we specifically transfect hundreds of clusters of cells with fluorescent reporter constructs and measure single-cell fluorescence changes using automated microscopy.

Reverse transfection arrays were first described as a method to introduce mammalian cDNA constructs into adherent cells at defined locations [5]. Since then, the method has been adapted considerably to improve performance and expand its range of applications [6-12]. As any transfectable molecule can be used in this system, reverse transfection studies have been published using cDNA [5], siRNA/shRNA [13-17] and reporter plasmids [6,18]. However, despite the enormous potential of this method, its use has mostly been centred on endpoint assays. Using a novel dual-fluorophore reporter construct, we studied the upregulation of three inducible promoters transfected in 600 independent clusters of cells. Automated image analysis allows us to segment single cells and quantify their normalized fluorescence intensity. The method is applicable to several cell-types and can be scaled for parallel expression measurements of hundreds of gene promoters. The Living Microarray provides an *in vivo *platform for studying complex transcriptional processes such as cell-cycle and cell differentiation, as well as establishing models of transcriptional stochasticity.

## Results

### Construction of the Living Microarrays

**Figure 1:**
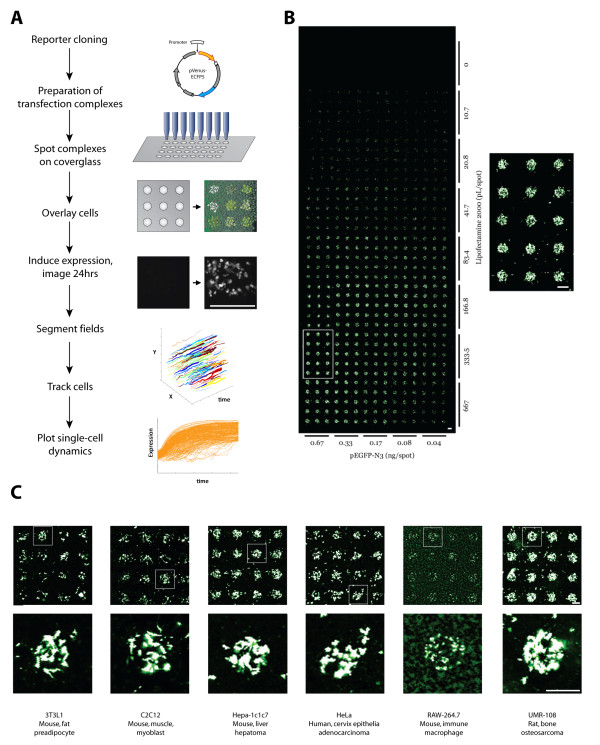
**Living Microarray method**. (A) Platform Workflow. The Living Microarrays method allows for highly parallelized measurement of reporter gene expression at the single-cell level. Synthetic promoter cassettes are cloned into the VC5 reporter construct and spotted with transfection reagent onto glass slides. Transfected cell clusters are imaged iteratively using automated microscopy and analyzed to generate normalized single-cell measurements of transcriptional activity. (B) Whole chip view of a Living Microarray. HEK-293T cells were reverse-transfected at 600 spots with varying ratios of pEGFP-N3 and Lipofectamine2000 and fixed cells were imaged at 10 μm resolution using a microarray scanner. Maximal transfection conditions are indicated by the white box and shown at higher magnification. (C) Reverse transfection reporter microarrays using human, mouse and rat cell lines originating from various tissues transfected with pEGFP-N3. Scale bars = 350 μm.

Living Microarrays use fluorescent reporter constructs to measure the expression of individual promoters in live cells. High throughput parallelization is achieved using a reverse transfection protocol [5], whereby transfection complexes containing reporter constructs are spotted at defined locations on a solid substrate. A monolayer of adherent cells is overlaid and cells adhering to a specific spot become transfected and express a fluorescent reporter protein under the control of the promoter of interest. Single-cell expression measurements are then made by segmenting imaged spots and quantifying the amount of intracellular fluorescence using automated image analysis tools (Figure 1a).

In our experiments, the array is created using spots with 6.7 nL transfection complex; these have a 400μm diameter and typically result in 75 transfected cells (293T cells). Using a square pattern we can spot 600-1000 spots within an 8.6 cm^2 ^chambered coverglass slide (Figure 1b), though this can easily be doubled by spotting in a checkerboard layout or using a substrate with a larger surface area. The higher throughput represents a significant improvement over existing high-content screening platforms. Moreover, since all transfections take place within a single chamber, variations due to plating density, media composition and drug concentrations can be minimized.

To test the suitability of our platform with other cell lines, we tested 14 adherent cell lines commonly used as tissue models for disease. We successfully transfected a constitutively active fluorescent reporter (pEGFP-N3) into lines originating from a variety of tissues, including fat, muscle, liver and bone (Figure 1c, Additional file 1, Table S1). The reverse transfected cells were spotted on standard glass slides, fixed and imaged on a conventional microarray slide scanner. Alternatively, our system uses automated microscopy to serially acquire high-resolution images of live cells at each transfected spot. The scan is repeated continuously to generate time-lapse videos of fluorescence changes for each transfected reporter construct.

While the positions of dried spots can be easily located on the microscope, these boundaries disappear once the slide has been flooded with cells. We therefore developed a method of registering slides on the microscope using the positions of dried spots relative to invariant features at the edges of the slide. To avoid using autofocus routines that are time-consuming and unnecessarily expose cells to potentially damaging light, we also developed a method to determine accurate focus positions for all 600 transfected clusters by fitting a subset of 45 manually-focused areas to a third-order polynomial function that closely approximates the surface of our slide (Additional file 1, Figure S1a). These 600 focused positions are automatically adjusted at every pass to correct for any drift during the acquisition (Additional file 1, Figure S1b). In this manner, we have been able to image each transfected cluster of cells every 20 minutes over periods as long as 7 days.

### Development of a dual-fluorophore reporter

**Figure 2:**
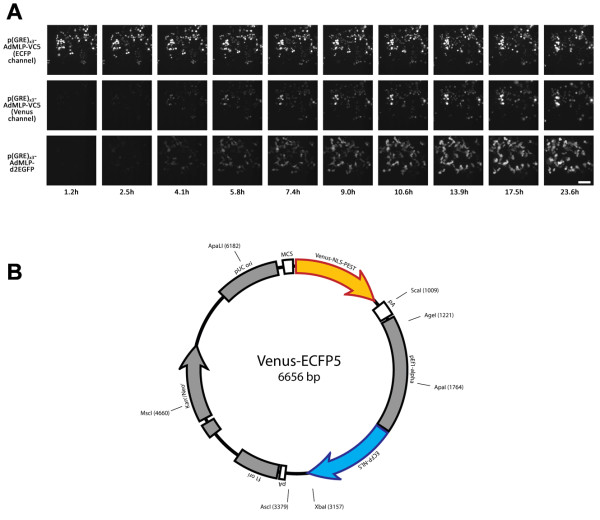
**Measurement of transcription using a dual-fluorophore vector**. (A) Time-lapse images of (GRE)_x3_-AdMLP induction by dexamethasone in 293T cells using nuclear and cytoplasmic reporters. The first two rows represent the same spot transfected with the p(GRE)_x3_-AdMLP-VC5 vector and imaged in ECFP (top) and Venus (middle) channels. The induction profile using the Venus reporter is comparable to that of the cytoplasmic destabilized EGFP reporter, p(GRE)_x3_-AdMLP-d2EGFP, shown in the bottom row. Moreover, the use of the ECFP channel enables the tracking of transfected cells before induction has taken place. Scale bar = 100μm. (B) Map of the Venus-ECFP5 (VC5) plasmid.

To measure dynamic changes in transcriptional activity, we used the Venus-NLS-PEST fluorescent reporter [19]. This reporter offers several advantages for measuring transcription. First, the Venus polypeptide matures 15 times faster than EYFP at 37°C and is 30 times brighter [20]. It is therefore well-suited for measuring dynamic expression changes over time without the long maturation time typically associated with fluorescent reporters. Second, the reporter is destabilized by fusion with the PEST domain of ornithine decarboxylase, such that it could also measure down-regulation of promoter activity. This modification has been previously shown to reduce the fluorescence half-life of EGFP to 2 hours in mammalian cells [21]. Finally, fusion of Venus to the SV40 large-T antigen nuclear localization signal (NLS) allows for signal to be restricted to the cell nucleus, whose regular shape is suitable for automated segmentation of the cells. When placed downstream of an inducible promoter consisting of a three-copy glucocorticoid response element (GRE) and the adenovirus major-late minimal promoter (AdMLP), we observed robust induction of Venus fluorescence that was comparable in intensity to a commercially-available cytoplasmic destabilized EGFP reporter (pd2EGFP-1, Figure 2a). However, the nuclear localization of the Venus signal allows single-cell intensities to be unambiguously determined, particularly for adjacent cells with overlapping cytoplasm (Figure 2a).

We automatically measure single-cell expression values by segmenting each frame to identify nuclear boundaries and measure the total pixel intensity within each segmented region (Additional file 1, Figure S2). Cells are identified using Watershed segmentation since it is computationally efficient [22] and performs better on our data compared to global thresholding and contour-based methods, particularly where cells in our images are clustered or the image background is uneven. To evaluate the performance of our segmentation and tracking algorithms, we used a film strip visualization with computationally annotated cell contours and manually examined images for 2340 single cells transfected with EF1α-VC5 over the 24 hour time course. Of these, 14 cells had one or more errors in segmentation or tracking (such as grouping multiple cells or identifying cells not present), which corresponds to an error rate of 0.6%.

To test our Venus reporter, we modified the well-known pGL3 reporter system by replacing the luciferase gene with a dexamethasone-inducible Venus-NLS-PEST ((GRE)_x3_-AdMLP-GV3). 293T cells were co-transfected with this plasmid and a second reporter containing a constitutively active, nuclear-localized dsRed (pCMV-dsRed-nuc) to control for transfection efficiency. Single-cell segmentation of cells imaged in Venus and dsRed channels allowed us to measure a normalized upregulation of cellular fluorescence of 11.0 fold (+/- 0.7) after 24 hours (Additional file 1, Figure S3a).

Conventional transfection-based reporter strategies typically rely on co-transfected plasmids to normalize signal for variations in plasmid copy number, for instance co-transfection of a plasmid constitutively expressing *Renilla *luciferase while the target signal is detected using *Photinus *luciferase. This approach has been previously used in reverse transfection arrays to normalize spot to spot differences of estrogen receptor alpha transcription in fixed MCF-7 cells [23]. However, our data indicates that it is not feasible to co-transfect a reporter plasmid to normalize gene expression in single cells, since it results in a variable partition of plasmids among cells. Even in highly transfectable cells (293T), co-transfection of our Venus-NLS-PEST reporter with CMV-dsRed-nuc resulted in only 17.6% (+/- 1.8) of image pixels having overlapping signal from both fluorophores (Additional file 1, Figure S3b).

We therefore cloned downstream of Venus a cassette containing ECFP driven by the human EF1α promoter (Figure 2b) and transcriptionally insulated the two genes using the poly-adenylation signal from the pGL3 vector. The EF1α promoter is constitutively active in mammalian cell lines, but it is not subject to spontaneous down-regulation like high-level viral promoters such as the CMV immediate early promoter-enhancer [24,25]. As with Venus, we fused ECFP to the SV40 NLS to allow for more accurate quantification of total cellular fluorescence. Information from this channel provides three major benefits. First, it allows us to measure relative differences in plasmid concentration between cells. Second, the constitutive ECFP signal is useful for segmenting and tracking cells in which Venus signal is expressed below detectable levels. Third, since the distribution of ECFP pixels is the same as for Venus, this channel allows us to normalize for other sources of variability, such as morphological changes and lamp fluctuations.

### Single-cell transcriptional measurements of inducible promoters

To test our system, we subcloned three inducible promoters into the multiple-cloning site of Venus-ECFP5 (VC5, Figure 2b). These promoters contain three copies of consensus response elements for the glucocorticoid receptor (GRE) or the retinoic acid receptor (RARE) cloned upstream of the AdMLP minimal promoter. A third reporter was also created using the tetracycline-inducible promoter from the commercially-available pTRE-Tight-Bi vector. Each construct, along with positive and negative controls, was reverse transfected in 75 replicate spots into 293T cells in triplicate assays. Following transfection, the cells were treated with the appropriate ligands and activity of the fluorescent reporters was measured over a 24 hour period. Each transfected cluster was imaged every 20 minutes, resulting in 600 time-lapse videos of 72 frames in length. Approximately 85 individual cells were automatically segmented and quantified in each image (over 11 million segmented cells in total) and were used to calculate average expression values for each construct over time.

**Figure 3:**
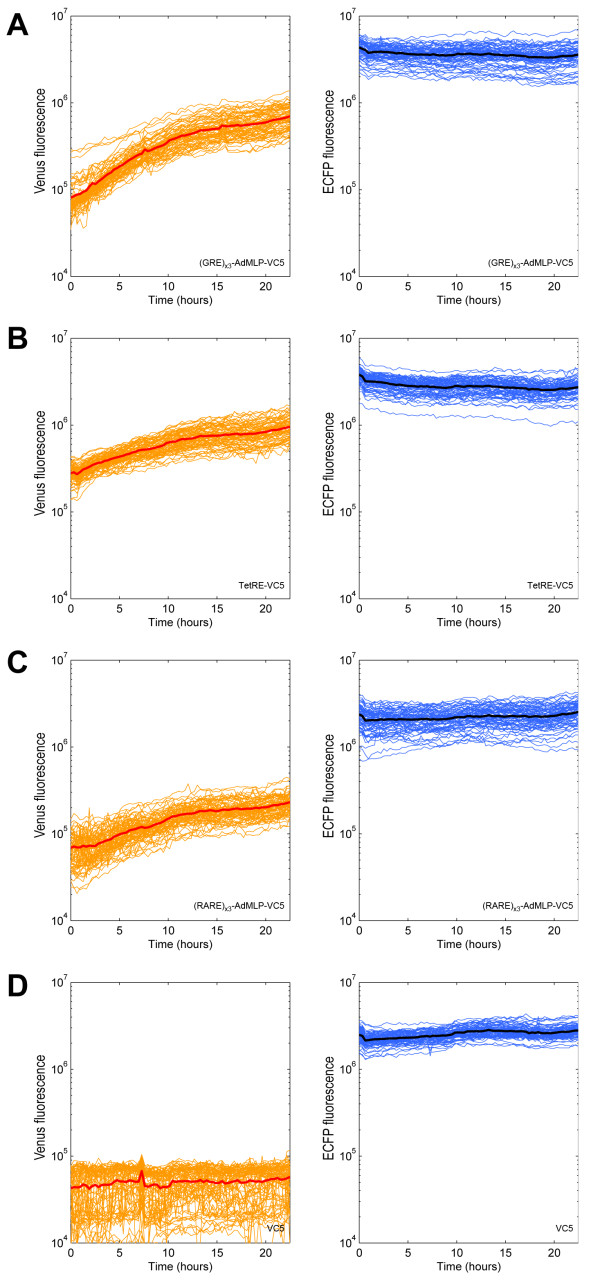
**Global expression changes**. 293T cells were reverse-transfected with inducible constructs, each arrayed over 75 replicate spots, and fluorescent cells were imaged over 24 hours. Single-cell measurements at each spot were averaged for the Venus (orange) and ECFP (blue) channels and are shown over time for (A) p(GRE)_x3_-AdMLP-VC5 following induction with 1 × 10^-7^M dexamethasone, (B) pTetRE-VC5 following induction with 2.2 × 10^-6^M doxycycline, (C) p(RARE)_x3_-AdMLP-VC5 following induction with 1 × 10^-6^M all-trans retinoic acid and (D) the promoterless VC5 reporter. The mean of all replicate fields is drawn in red for Venus signal and black for ECFP signal.

We observed induction of Venus transcription for all three synthetic reporter constructs, while the expression levels from the normalization channel remained relatively constant (Figure 3). The (GRE)_x3_-AdMLP-VC5 construct was upregulated 11.9 fold (+/- 4.9) over 24 hours (Figure 3a, Additional file 1, Figure S3c) which is consistent with the published literature [26,27]. This level of induction matches that measured in our single-fluorophore vector (GRE)_x3_-AdMLP-GV3, demonstrating that the further modifications brought about in VC5 do not alter reporter activity (Additional file 1, Figure S3). In comparison, our promoterless vector (VC5) showed no change in expression over 24 hours (Figure 3d). Efficient induction was observed for both the (GRE)_x3 _and TetRE reporters when cotransfected with their respective transcription factors (which are not expressed endogenously in 293T cells), as well as for the (RARE)_x3 _reporter, which is regulated by endogenously expressed retinoic acid receptors.

Our measurements of gene expression were obtained using data from 75 replicate spots. To determine the minimum number of replicate spots that would accurately measure the sample mean and variance, we used a permutation strategy in which the data was segregated into subsets of a fixed number of randomly chosen spots and repeated this process 1000 times for subsets of between 1 and 20 spots. The distribution of means from these subsets at the 15 hour timepoint was then compared to the actual distribution for 75 transfected spots. We found that samples with 5 replicates had a standard deviation that was within 0.5 standard deviations of the population standard deviation.

**Figure 4:**
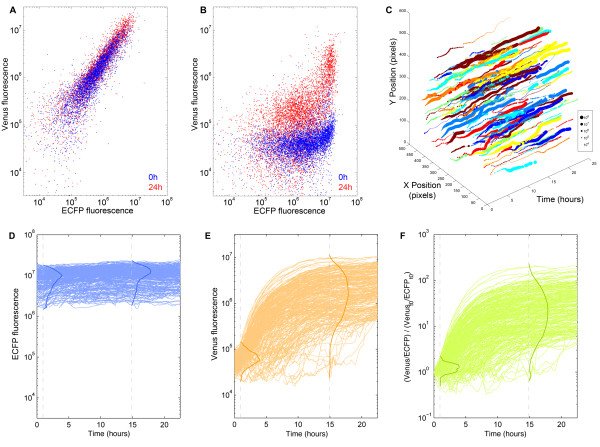
**Single-cell measurements of promoter activity**. Single-cell measurements are plotted at 0 (blue) and 24 hours (red) post-induction for (A) EF1α-VC5 and (B) p(GRE)_x3_-AdMLP-VC5. (C) Multi-dimensional view of inducing cells on a single spot. Spatial movement of cells from one field is shown where each cell is represented by a different color and the intensity of normalized Venus fluorescence is proportional to the size of the dot. (D-F) 293T cells transfected with p(GRE)_x3_-AdMLP-VC5 were tracked over 24 hours after induction with 1 × 10^-7^M dexamethasone. Fluorescence measurements for single cells having induced over 3 standard deviations above the initial mean are displayed for (D) ECFP and (E) Venus. (F) Single-cell expression profiles normalized for ECFP signal. Darker lines represent the distribution of normalized cell intensities at 1 and 15 hours following treatment, illustrating cellular heterogeneity in the transcriptional response.

These endpoint measurements are analogous to existing methods that average responses between cells, such as RT-PCR, DNA microarrays and reporter assays. However, in addition to being able to measure average expression changes, single-cell measurements provide a wealth of information that can be used to better characterize signal variability between cells. Using the signal from our ECFP cassette, we were able to study the single-cell distribution of responses with respect to transfection efficiency (Figure 4a-b). Data from the EF1α-VC5 positive control, where each fluorophore is driven by the EF1α promoter, showed that signals from both fluorophores are correlated (R^2 ^= 0.73 - Figure 4a), indicating that cells transfected with more copies of plasmid (determined by the ECFP level) show a greater level of reporter fluorescence. Among the inducible constructs, the ECFP signal allowed us to quantify the relationship between plasmid copy number and peak reporter activity in inducing cells (Figure 4b). Our data suggests that there exists a large degree of signal variability underlying each static point obtained from pooled methods. For instance, although the average Venus induction in cells transfected with (GRE)_x3_-AdMLP-VC5 was 11.9 fold, segmentation of 6,339 transfected cells indicates that 3,532 cells (55.7%) induced less than 3 fold, while a small number of cells (523 cells - 8.3%) showed very large responses (over 50 fold). This observation was reproducible across three separate passages of cells (Additional file 1, Figure S4).

### Single-cell tracking of inducing cells

The single-cell population endpoint measurements could also be obtained using other technologies, including two-color flow cytometry. A distinct advantage of the Living Microarray system is that expression changes in individual cells can be tracked over time by linking single cells in consecutive frames. We observed that fluorescent proteins tend to concentrate within sub-nuclear regions, such that each cell has a characteristic appearance that remains relatively constant between frames. We exploited this feature to track each cell over time based on its appearance and position (Additional file 1, Figure S5). More dramatic changes in appearance, such as when cells are occluded, are handled by a flexible linking step that joins tracks based on cell position and intensity. Since our experiments typically generate large amounts of data (over 100 Gb per experiment), we implemented our segmentation and tracking algorithms to analyze each field separately, such that computation time can be shortened by using parallel computing.

These steps result in a set of tracked cells per field, where each track contains information about the cell's position, intensity and shape over time that can be visualized in many ways. For instance, each cell's position and intensity from a single field can be imaged as 3D trajectories inside a spot-centred space-time volume (Figure 4c, Additional file 1, Figure S6), such that the dynamics of single-cell expression changes can be viewed in relation to its mobility, its division events or its neighbours. Among cells that showed the strongest induction (greater than 3 standard deviations from the initial mean Venus fluorescence), we observed 10-fold greater induction compared to pooled measurements (Figure 4e compared to Figure 3a) with coefficients of variation of 8.50% for (GRE)_x3_-AdMLP-VC5 (Figure 4e) and 5.60% for (RARE)_x3_-AdMLP-VC5 (Additional file 1, Figure S6b). Moreover, single cells with different levels of transfection can be compared by normalizing the Venus signal with signal from the ECFP channel (Figure 4d-f, Additional file 1, Figure S6). Among cells that showed the strongest induction (greater than 3 standard deviations from the initial mean Venus fluorescence), this resulted in an expression plot with a shape and distribution similar to the uncorrected Venus signal. However, normalizing the signal for transfection efficiency resulted in a change in the rank order of the cells at the 24-hour timepoint, such that poorly transfected cells were prioritized.

**Figure 5:**
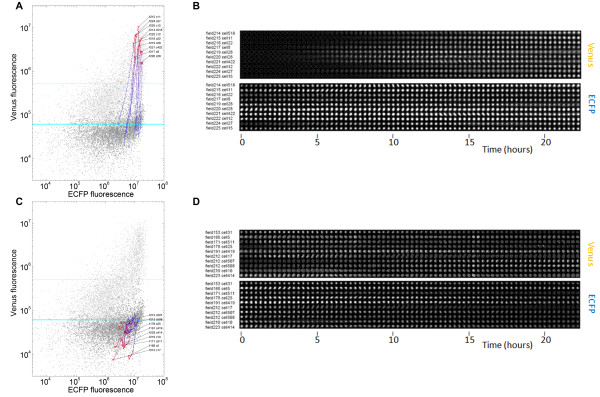
**Single-cell tracking identifies different profiles of transcriptional activation**. 293T cells transfected with p(GRE)_x3_-AdMLP-VC5 were induced with 1 × 10^-7^M dexamethasone and tracked over 24 hours. (A, C) Tracks of cells inducing (A) and remaining unchanged (C) over 24 hours. Each cell track is represented by a line where each dot represents a different timepoint and the color describes the time of induction from 0 (blue) to 24 hours (red). The lower teal line represents the mean of the starting population, while the upper teal line shows the mean + 3 standard deviations. Single-cell time-lapse images are shown for cells that have similar starting ECFP expression levels, but whose response to dexamethasone is either high (B) or remains unchanged over time (D).

The ability to track single cells allows us to extend population measurements by evaluating the dynamics with which a given cell will change its expression in response to a stimulus (Figure 5a). Moreover, our image-based assay can recover the appearance of the cells over time and display these as single-cell image strips. While we had observed substantial heterogeneity in the extent of the response to induction, this view shows additional variation in the timing of Venus expression among cells that fully induced (Figure 5b). Compared to methods that only examine responding cells, our platform enables each inducing or un-inducing cell's history to be viewed in relation to its size and morphology (Figure 5b-c). This is a feature that could be particularly powerful when examining processes involving changes in cell shape, such as cell division and differentiation.

## Discussion

We have described the development of a high-throughput platform capable of measuring transcriptional dynamics for many genes in large numbers of single cells exposed to the same experimental conditions. Existing methods for measuring gene regulation, such as DNA microarrays and chromatin immunoprecipitation, have excelled at identifying members of transcriptional networks and the interactions that occur between them at specific points in time. However, these techniques acquire data from homogenized samples derived from populations of cells and cannot provide accurate temporal and spatial resolution at a scale appropriate to characterize time-dependent transcriptional responses [28,29].

Here we used a high-throughput cell-based assay to specifically transfect hundreds of constructs into adherent cell lines and tracked each fluorescent cell over 24 hours. We adapted the technique to use reporter constructs, so that changes in transcription from different promoters could be assessed at single-cell resolution. In its current form, 600 spatially distinct clusters of cells can be specifically transfected in a single chamber. This removes many sources of well-to-well variability that are frequently associated with high-content screening, such as differences in seeding density, ligand concentration and temperature.

Using the ECFP channel provides an internal control that enables single-cell normalization of the target promoter activity levels without requiring co-transfection. Tracking uninduced cells was also possible using this channel, such that we could investigate each cell's history during the experiment. As with the Venus reporter, destabilizing the ECFP protein by fusing it to a PEST domain could provide a more sensitive method of detecting loss of the plasmid and relating this to variability in the Venus channel.

Despite identifying several cell lines suitable for our platform, we determined that a subset of lines (such as F442-A, AtT-20, MCF-7 and HepG2) either did not grow as monolayers or could not be transfected at high enough levels to be used (Additional file 1, Table S1). Viruses offer an attractive alternative to transfection-mediated delivery of DNA into cells that are difficult to transfect, particularly primary cells. Previous studies using VSV-G lentiviruses [10] and more recently adenoviruses [11] have demonstrated the feasibility of the reverse infection approach that would be compatible with our live cell techniques. Expressing the reporter from a single integration site would provide the added benefit of removing reporter variability from differences in plasmid copy number between cells. Moreover, adenovirus systems can accommodate larger insert sizes compared to retroviral ones, such that our dual fluorophore vector could be re-used.

The measurement of single-cell expression offers much potential and has been studied using other methods, such as the dynamic proteomics approach [30] where coding sequences are randomly fused to an EYPF cassette in their endogenous context. The primary advantage to this technology is that expression can be studied from a single-copy integrant that would presumably retain the endogenous regulation of the gene at both the transcriptional and translational levels. However, a significant disadvantage of the method is that specific genes cannot be targeted using this method, as it generates a library of randomly integrated YFP cassettes that subsequently requires extensive screening. The maintenance of multiple cell lines, each harbouring a different YFP-tagged gene, is also a limitation that is addressed in our method, where the expression of hundreds of genes can be studied in parallel within a single well.

The Living Microarray platform can be applied to many systems. Expanding the number of synthetic reporters could provide parallel measurements for the activities of dozens of transcription factors in response to various stimuli, as has been shown with pooled measurements of sequencing and luciferase-based reporters [27,31]. The technology could also be used to identify genomic regulatory sequences. Given the high-throughput nature of this technology, it would be possible to screen large regions of non-coding DNA for transcriptional activity by generating a library of reporters from tiled PCR products. In particular, this approach could be useful for validating putative regulatory variants from genome-wide association studies. Constructing a Living Microarray with known promoters could also profile complex processes, for example in studying the transcriptional changes that occur during the cell cycle [32-34], where temporal profiling of transcription in mammalian cells has been limited to microarray analysis using pooled RNA from synchronized cultures [35-37]. Such studies suffer from limited time resolution and fail to capture cell-cell variability or the heritability of expression patterns over successive generations. Moreover, chemical synchronization of cells may introduce spurious expression patterns in addition to only being partially effective [38]. A recent report performed genome-wide siRNA knockdowns in reverse transfection microarrays and scored the phenotypes of the transfected clusters by computationally classifying time-lapse images of cytoplasmic and nuclear fluorophores during mitosis [17]. In this way, hundreds of genes were identified as being required for the cell cycle. An attractive extension to these findings would be to construct a Living Microarray with reporters for these genes to precisely map their temporal regulation.

Gene expression is an inherently stochastic process, both within single cells and among cells of a population, owing to the many sources of intrinsic and extrinsic noise [39,40]. For instance, clonal populations of mouse haematopoietic stem cells display heterogeneity in transcriptional profiles; these differences in genetically identical cells are responsible for each cell's propensity to differentiate into myeloid or erythroid lineages [41]. Cell-fate decisions, such as pheromone switching in isogenic yeast, can also arise from heterogeneity that is more related to the individual cell's signal transmission and expression capacities, rather than on random fluctuations in gene expression [42]. Nuclear receptor signaling is an ideal model for studying dynamic transcriptional processes, since the timing of induction can be tightly controlled. We found considerable variability in response at the single-cell level that is consistent with previous reports using single-cell measurements. For instance, reporter studies on the GH gene promoter revealed that when activated, only 25% of cells displayed a sustained response, while 50% showed only a transient one and 25% were not induced at all [43]. Single-cell studies of the prolactin promoter in pituitary cells have indicated that the apparently stable transcription rate in a population may represent the overall sum of dynamically variable patterns of promoter activity among the individual cells [44-46]. This suggests that within populations of cultured cells (and perhaps normal tissues), there exists a mixture of cells that have different capacities to respond to external stimuli. Whether this heterogeneity reflects the presence of distinct subpopulations of cells, or results from normal fluctuations in cell physiology (possibly resulting from changes in cell cycle or metabolism) are questions that merit further investigation.

## Conclusions

The advent of high-throughput methods for measuring changes in gene expression has facilitated the study of biological systems as a whole (i.e. gene interaction networks), rather than on the discovery and characterization of its individual components (i.e. genes) [47]. However, deep understanding of a complex dynamic system requires an examination of the dynamics of individual components during normal function or following perturbations [48]. The Living Microarray platform is a step towards creating a more comprehensive platform for furthering our understanding of dynamic cellular processes at the systems level, as it provides the ability to make parallel high-throughput measurements of transcriptional changes in single cells.

## Methods

### Plasmid construction

A detailed description of the cloning steps involved in plasmid construction is contained in Additional file 2.

### Reverse transfection

Transfection complexes containing reporter plasmid, transfection reagent and fibronectin were prepared as previously described [5,7]. Briefly, 500 ng total plasmid was mixed with 0.5 μL Lipofectamine 2000 (Invitrogen, Burlington, ON) in OptiMEM (Invitrogen, Burlington, ON - total volume 12 μL) and allowed to incubate for 20 minutes at room temperature. 3 μL of a 0.1% fibronectin solution (Sigma-Aldrich, Oakville, ON) was added before transferring the complexes to a 384-well plate. Transfection complexes are arrayed on chambered coverglass slides (Nunc, Rochester, NY) using a GeSim nanoplotter equipped with Nano piezo-tips. Twenty drops of transfection complex are dispensed at each spot, yielding a spot diameter of 400 μm formed with 6.7 nL of transfection complexes. Spots are spaced 900 μm apart from their centers. Our experiments involved spotting 75 replicates for each construct for a total of 600 spots per array. For transfections involving GRE or TetRE reporters, we also co-transfected 50 ng of a plasmid constitutively expressing glucocorticoid receptor or TetOn Advanced (Clontech, Mountain View, CA), respectively. Three replicate arrays were incubated in a vacuum desiccator for at least 1 hour before plating cells. 293T cells were cultured in DMEM containing 10% heat-inactivated, charcoal/dextran-treated fetal bovine serum (stripped serum - HyClone, Logan, UT) and 1 × 10^6 ^cells were plated in antibiotic-free medium and allowed to settle on the spots overnight at 37 degrees. Reporter constructs were induced 16 hours following transfection by changing the medium to DMEM containing 10% stripped serum with antibiotics and 1 × 10^-7^M dexamethasone (Sigma-Aldrich, Oakville, ON), 1 × 10^-6^M all-trans retinoic acid (Biomol International, Plymouth Meeting, PA) and 2.2 × 10^-6^M doxycycline (Sigma-Aldrich, Oakville, ON).

### Standard transfection

Transfections were performed in triplicate in 6-well dishes (Corning, Lowell, MA) seeded the previous day with 1 × 10^6 ^293T cells grown in stripped serum. Transfection complexes were prepared with Lipofectamine 2000 (Invitrogen, Burlington, ON) as per the manufacturer's instructions, except that we used half the recommended amount of transfection reagent. Cells were treated with 1 × 10^-7^M dexamethasone (Sigma-Aldrich, Oakville, ON) and were incubated for 24 hours at 37 degrees before imaging.

### Imaging setup

Imaging of the Living Microarray is performed using an inverted fluorescence microscope (TE2000, Nikon, Melville, NY) with a 20X objective (numerical aperture = 0.75) and an ultra stable close-loop feedback mercury light source (X-Cite exacte, EXFO, Mississauga, ON). The microscope is equipped with an incubator maintaining the cells in a humidified environment at 37°C and 10% CO_2 _(Solent Scientific, Segensworth, UK). The motorized stage includes encoders with accuracies of 0.5 μm and 0.02μm in the X/Y and Z axes, respectively (ProScan™II, Prior Scientific, Rockland, MA). Fast switching (<50 ms) excitation and emission filter wheels used in combination with a multiple band dichroic mirror (filter set 86006, Chroma, Bellows Falls, VT) allows for rapid switching between channels. Image capture is performed using a back-thinned electron multiplier CCD camera (C9100-12, Hamamatsu, Bridgewater, NJ). A custom adapter was designed to hold the Labtek Chamber slides onto the stage. The stage, filter wheels and camera are controlled using custom software based on the micro-manager package http://www.micro-manager.org.

### Slide registration

Deviations in printed spot positions are detected by imaging each printed slide using a digital camera. After correcting the image for lens distortion (pincushion type) as previously described [49], we determine the location of each spot. The image is convolved with a disk shaped operator of equal diameter to each spot before applying a Watershed transformation (MATLAB). The exact location of the spot centres is determined by calculating the intensity-weighted centroid of each segmented region. These spot centres are then mapped to microscope stage coordinates using the stage positions of three spots at the corners of the array. The coordinates of two invariant features on the edge of the slide are also used to calculate the position of each transfected cluster in the array.

### Focus interpolation and acquisition settings

The focus positions of 45 spots evenly distributed across the array are manually determined. The focus positions (z) of the remaining spots are subsequently interpolated by fitting these 45 coordinates to a third degree polynomial surface:

$$z=\sum_{i=0}^{3}\sum_{j=0}^{3}a_{ij}x^{i}y^{j}$$

We used RANSAC outlier detection for added robustness. After each pass of acquisition, these focus positions are corrected using a global offset estimated from autofocused features. The autofocus routine uses an autocorrelation-based focusing algorithm [50] over four invariant features located under the chamber slide wall. A global focus offset is calculated from the median drift for the four focus positions and applied to all spots.

In our experiments, each field was imaged once with the ECFP filter (gain: 90; exposure: 0.027 s) and at two different EYFP gain settings to maximize the dynamic range of the system (gain: 60, 120; exposure: 0.027 s). Each spot was therefore exposed for at most 150 ms, minimizing fluorescence-induced cytotoxicity. One pass of 600 spots took 20 minutes and cells were imaged in this manner for 24 hours in three independent replicate experiments.

### Image Segmentation and Tracking

Watershed segmentation is prone to over-segmentation when applied to raw images. To avoid this we filter the raw images using a Fast-Fourier Transform in combination with a disk shape frequency domain filter [51]. We determined that a circular low-pass filter with a diameter of 69 pixels preserved nuclear shape while removing camera noise as well as variations in fluorescence within the nucleus. We then calculate the Watershed transform from the filtered image using an eight-connected pixel neighbourhood. This segmentation generates a segmented image where each segment, or group of pixels, either maps to a cell or a patch of background. We reject low intensity background segments, as well as small object segments with an area below 125 squared pixels (80 μm^2^). The low intensity segmentation constraint is calculated using the image histogram and set at 2000 units above the location of the first image peak. Single-cell fluorescence is quantified by summing the pixels within each cell's segmentation boundary. For each field we calculate background fluorescence as the mean of the lowest 10% cells and subtract this value from each cell's fluorescence.

Cell tracking is accomplished using a two step approach. The first step (strict linking) links two cells from consecutive frames that are less than 10 pixels (8 μm) apart and that have similar appearances. We calculate the appearance score as the average squared difference between normalized cell images (between 0 and 1) and minimize this score by rotating the images relative to each other. The chosen score threshold where cell links are kept is 0.0155. In the cases where multiple putative links have acceptable distance and appearance changes, the link with the best score is chosen. No link is established if there are discrepancies in the scores.

The second phase (flexible linking) establishes potential links between tracks from the first step based on distance. We determine the distribution of intensity differences between potentially linked cells across the slide and remove links where this difference is greater than 3 standard deviations. Links with the smallest intensity differences are then kept. Additionally, to allow for transient loss of tracking, for instance because a cell leaves the field or is occluded by another cell, we iteratively consider links over five successive time frames.

## Authors' contributions

SR conceived, designed and carried out all biological experiments and optimization, partly wrote the software for hardware control, generated some of the figures and wrote the manuscript. HD assembled and in some cases built the hardware, performed the microarray spotting, developed and implemented the routines relating to slide registration, focus interpolation and image analysis, generated some of the figures and edited the manuscript. HCPD cloned a subset of the reporter constructs and performed the Venus-dsRed validation experiments. RS and TJH contributed to the study design and provided supervision, intellectual contribution and manuscript revisions. All authors read and approved the final manuscript.

## Supplementary Material

Additional file 1 **Supplementary Data**. Contains additional figures and tables referenced in the manuscript. Click here for file

Additional file 2 **Supplementary Methods and Data (Plasmid Construction)**. Contains methods used to construct the plasmids used in the manuscript, including vector maps. Click here for file
