# Supplementary material for: The living microarray: a high-throughput platform for measuring transcription dynamics in single cells
Source: BMC Genomics. 2011 Feb 16;12:115. doi: 10.1186/1471-2164-12-115 (PMC3050818; doi:10.1186/1471-2164-12-115)
Supplement: Additional file 1 — Supplementary Data. Contains additional figures and tables referenced in the manuscript. [file 1471-2164-12-115-S1.PDF]

Supplementary Data

"The Living Microarray: a High-Throughput Platform for Measuring Transcription Dynamics in Single Cells". Rajan S, Djambazian H, Chu Pham Dang H, Sladek R and Hudson TJ

Figure S1: Focus correction. (A) Initial array-wide focus interpolation. Red dots represent 45 manually-focused spots from which the 600 focus positions (blue dots) and the scan path (blue lines) are interpolated using a third-order polynomial equation (see methods). (B) Compensation for focus drifts during the experiment. Focus drift is shown with respect to the initial focus position. The global focus offset is calculated from the median of 4 auto-focused regions after each pass.

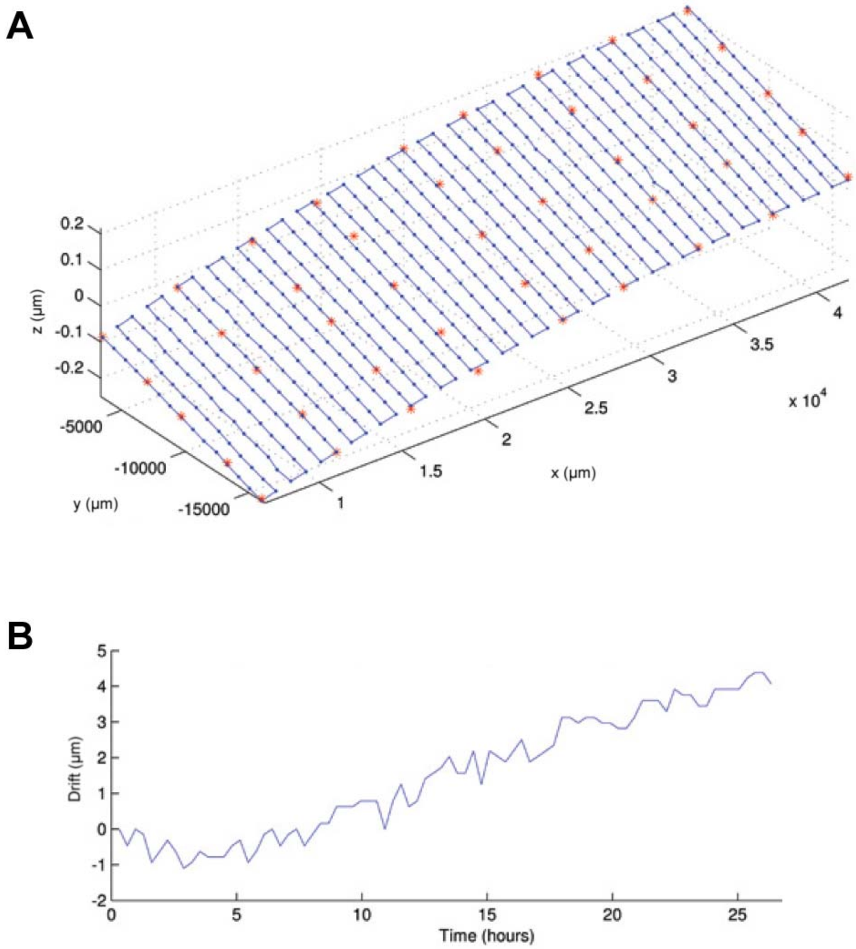

Figure S1: Automatic segmentation of nuclei. 293T cells were segmented on the ECFP channel to identify transfected cells. Fluorescent images were analyzed using a Fast-Fourrier Transform to establish a low-pass threshold used to filter the images. The filtering avoids over-segmenting the images by removing variability from the background and intra-nuclear regions. (A) original image; (B) FFT of raw image; (C) FFT of image after applying the low-pass threshold; (D) low-pass filtered image; (E) synthetic image showing segmented regions; (G) original image with segmented boundaries displayed. Scale bar = 100µm.

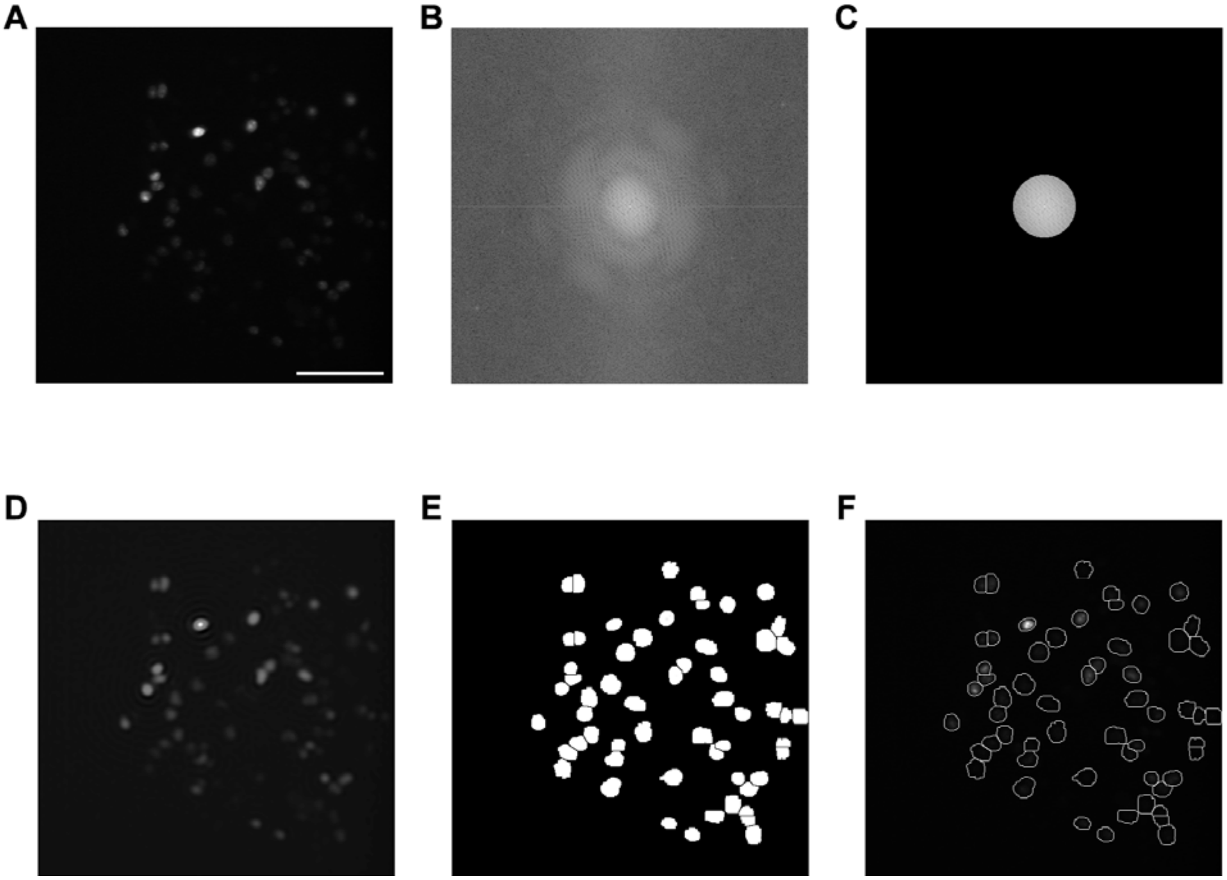

Figure S2: GRE inducibility of different reporters. Average normalized expression values were calculated from single-cell segmentation data of 293T cells transfected with GV3 and VC5 based vectors and induced with 10^{-7}M dexamethasone. (A) $(GRE)_{x3}$-AdMLP-GV3 co-transfected with CMV-dsRed-nuc; (B) Venn diagram depicting the amount of co-localized Venus and dsRed pixels in cells transfected with $(GRE)_{x3}$-AdMLP-GV3 after induction; (C) $(GRE)_{x3}$-AdMLP-VC5 reverse transfected over 75 independent spots.

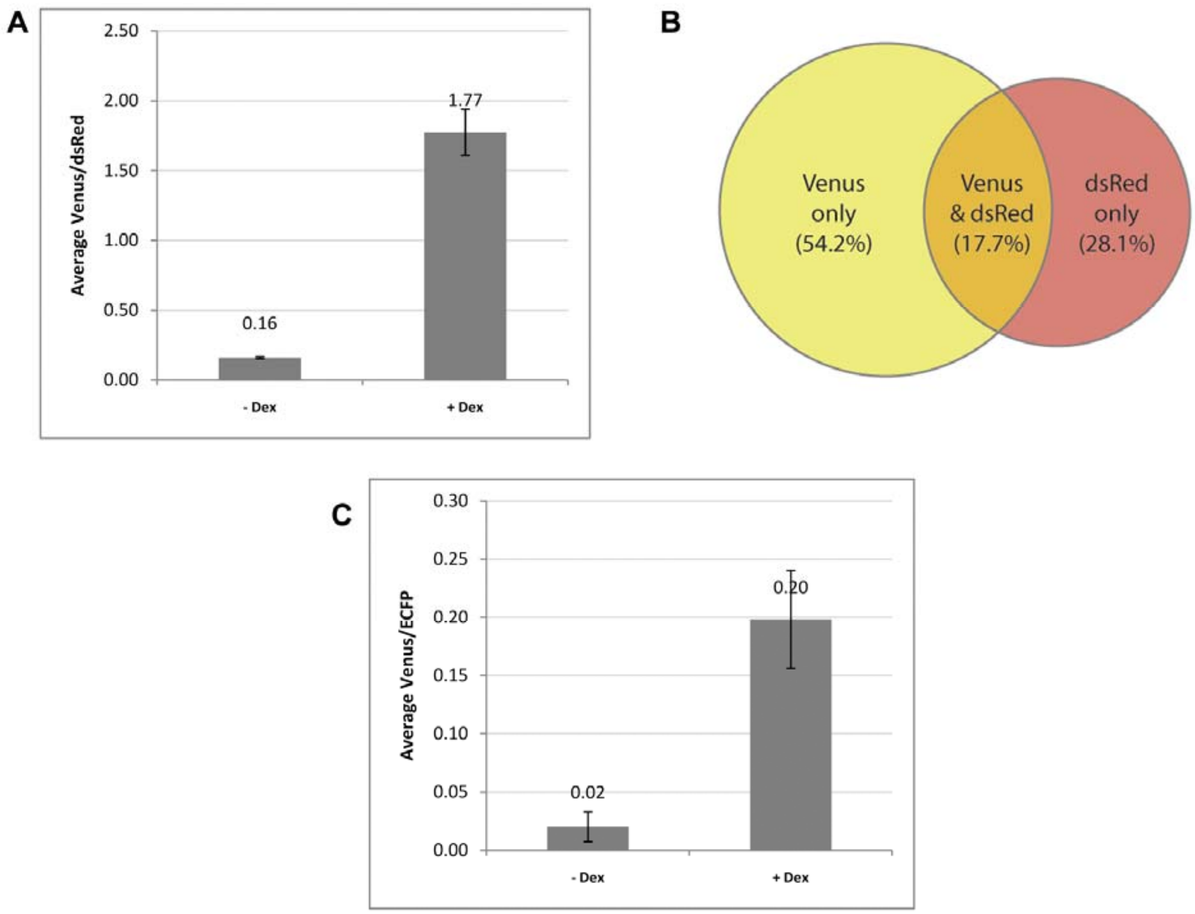

Figure S3: Replication of GRE induction. 293T cells were reverse transfected with $(GRE)_{x3}$-AdMLP-VC5 and single-cell intensities for Venus and ECFP are shown after 24 hours of induction as a density map. The experiment was replicated over three successive passages of cells (A-C). The blue line represents the mean Venus fluorescence at the initial timepoint, while dashed lines delineate the 3-fold and 50-fold induction thresholds. FC = Fold Change.

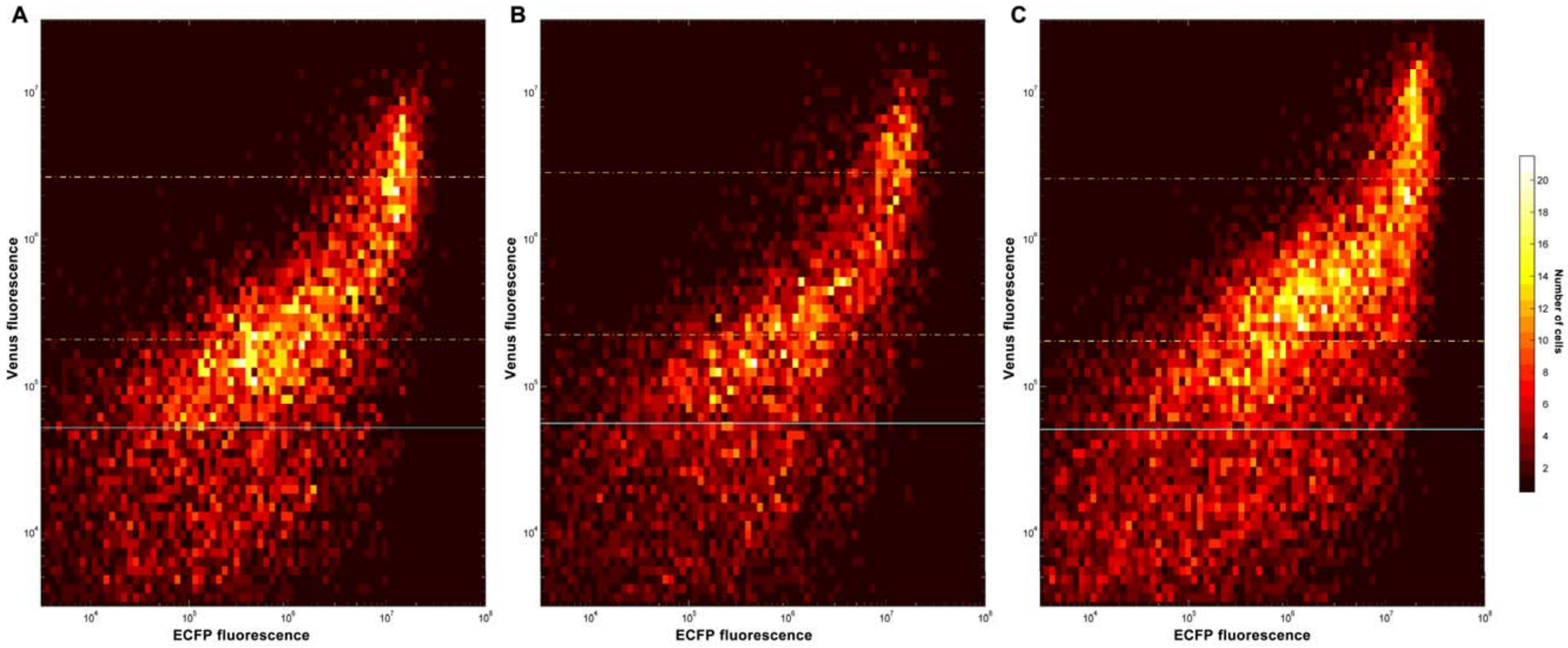

	Number	Percent
FC < 3	3,850 cells	56.3%
FC > 50	481 cells	7.0%
All cells	6,844 cells	100%

	Number	Percent
FC < 3	3,532 cells	55.7%
FC > 50	523 cells	8.3%
All cells	6,339 cells	100%

	Number	Percent
FC < 3	5,756 cells	50.4%
FC > 50	1,034 cells	9.1%
All cells	11,430 cells	100%

Figure S4: Cell tracking algorithm. (A) Two linking steps are used to track cells over long periods of time. The first step ("strict linking") links cells in successive frames that are within boundaries of distance, intensity and appearance. The second step ("flexible linking") links successive tracks using only distance and intensity parameters with less stringent thresholds. (B-C) Histograms of cell-cell links between successive frames measuring the changes in cell appearance, calculated as the normalized sum of squared difference values (B), and from changes in cell intensity (C). The red asterisk indicates the threshold set at two standard deviations from the mean.

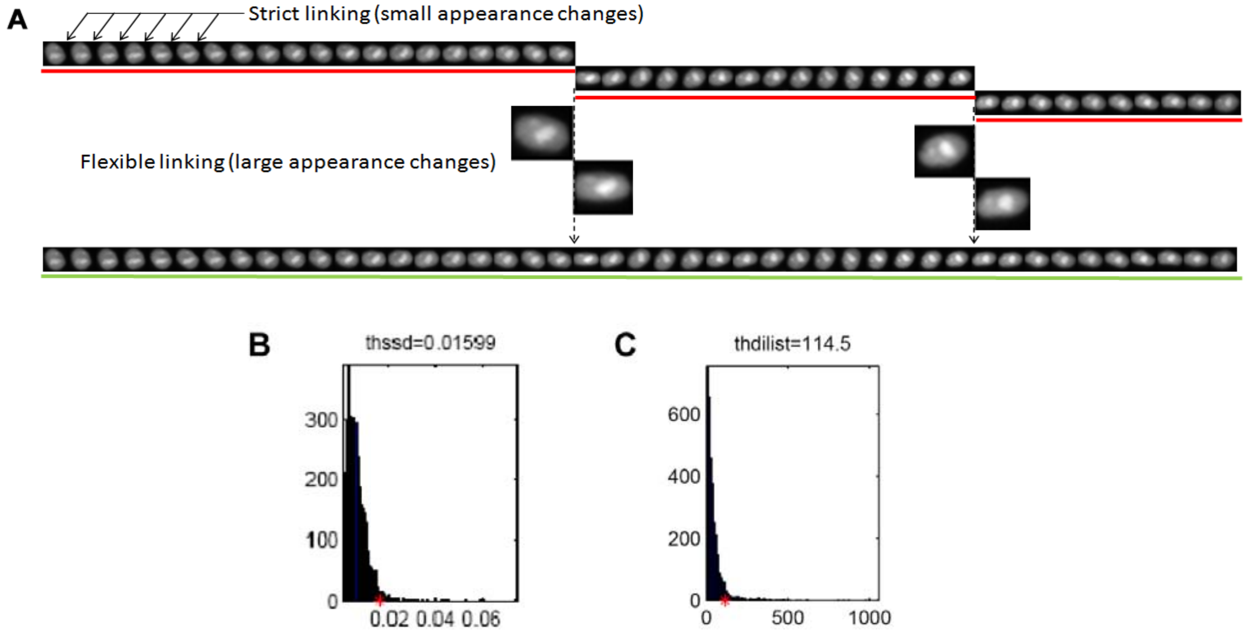

Figure S5: Single-cell measurements of promoter activity. Single-cell measurements for the p(RARE)$_{x3}$-AdMLP-VC5 reporter. (A-C) 293T cells transfected with p(RARE)$_{x3}$-AdMLP-VC5 were tracked over 24 hours after induction with $1x10^{-6}$M all-trans retinoic acid. Fluorescence measurements for single cells having induced over 3 standard deviations above the initial mean are displayed for (A) ECFP and (B) Venus. (C) Single-cell expression profiles normalized for ECFP signal. Darker lines represent the distribution of normalized cell intensities at 1 and 15 hours following treatment, illustrating cellular heterogeneity in the transcriptional response. (D) Multi-dimensional view of inducing cells on a single spot. Spatial movement of cells from one field is shown where each cell is represented by a different color and the intensity of normalized Venus fluorescence is proportional to the size of the dot.

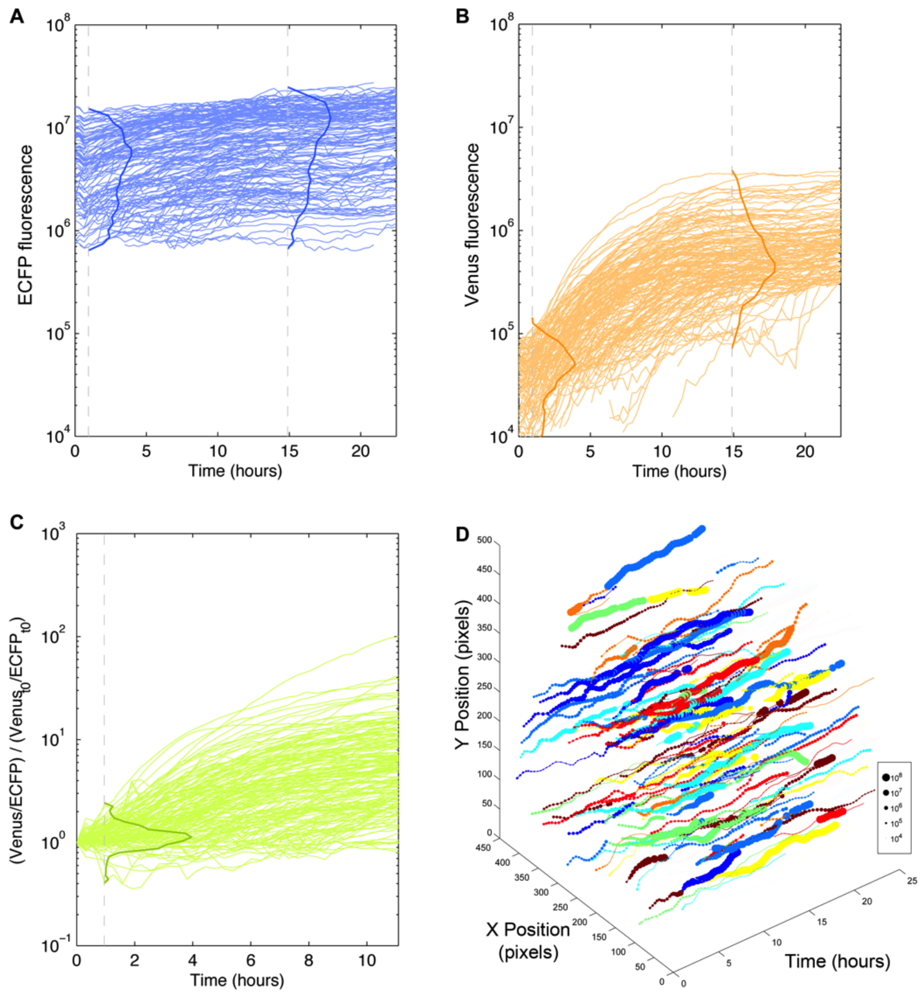

Table S1: Cell lines tested for transfectability on the Living Microarray.

Name	ATCC cat no	Organism	Tissue	Reverse transfection	Issue
**293T**	CRL-11268	Human	Embryonic kidney	yes	
**C2C12**	CRL-1772	Mouse	Muscle	yes	high mobility
**3T3-L1**	CRL-173	Mouse	Adipose	yes	high mobility
**NIH-3T3**	CRL-1658	Mouse	Fibroblast	yes	
**HepG2**	HB-8065	Human	Liver	no	does not form uniform monolayers
**AtT-20**	CCL-89	Mouse	Pituitary	no	Lipofectamine2000 toxic to cells
**F442A**	RefA	Mouse	Adipose	no	Poor transfection
**S16**	CRL-2941	Rat	Sciatic nerve (Schwann cell)	no	Poor transfection
**Hepa1c1c7**	CRL-2026	Mouse	Liver	yes	
**HeLa**	CCL-2	Human	Cervix (epithelial)	yes	
**Raw 264.7**	TIB-71	Mouse	Ascite (macrophage)	yes	
**Min6**	RefB	Mouse	Pancreas	no	Poor transfection
**UMR-108**	CRL-1663	Rat	Bone	yes	
**MCF7**	HTB-22	Human	Mammary gland (epithelial)	no	does not form uniform monolayers

A. Greene, H. and Kehinde, O. Spontaneous heritable changes leading to increased adipose conversion in 3T3 cells. *Cell* **7**, 105-113.

B. Ishihara, H. et al. Pancreatic beta cell line MIN6 exhibits characteristics of glucose metabolism and glucose-stimulated insulin secretion similar to those of normal islets. *Diabetologia* **36**,1139-1145 (1993).

Table S2: PCR primers used in vector cloning.

Primer name	Sequence
MCS_fwd	CGCGGCCGCTTAATTAAGTTTAAACGCGATCGCG
MCS_rev	GATCCGCGATCGCGTTTAAACTTAATTAAGCGGCCGCGGTAC
GRE-3C_fwd	TCGACAGGACAGCCTGTCCTCGCGGGTACAGCCTGTTCTGACTCGAGGACAGCCTGTCCTGGTAC
GRE-3C_rev	CAGGACAGGCTGTCCTCGAGTCAGAACAGGCTGTACCCGCGAGGACAGGCTGTCCTG
AdMLP_fwd	CGGGGGGCTATAAAAGGGGGTGGGGGCGTTCGTCCTCACTCTAGATCTGCGTTT
AdMLP_rev	AAACGCAGATCTAGAGTGAGGACGAACGCCCCCACCCCCTTTTATAGCCCCCCGGTAC
Ef1a-1206_left	GGTTATTAATGGAGAAGAGCATGCGTGAG
Ef1a-1206_right	GGTTGATATCTCACGACACCTGAAATGGAA
NLS-BsrG1-left	GCTATGTACAAGAGATCTCGAGCTGATCC
NLS-Not1-right	GTCAGCGGCCGCATCCTACCTTTCTCTTC
VenusNLS-left	TGCAGGATCCACCATGG
VenusNLS-Nhe-Asc-EcoRV-right	GCGTGATATCGGCGCGCCGCTAGCGGCCCAATGCATTGGC
Ef1a-nhe-left	AGTCGCTAGCGGAGAAGAGCATGCGTGAG
Ef1a-asc-right	CGTAGGCGCGCCGATGAGTTTGGACAAACCACAACTA
pA-nhe-left	AGTCGCTAGCAAGGTACGGGAGGTACTTGGA
pA-nhe-right	CGTAGCTAGCAGAGAAATGTTCTGGCACCTG
bRARE-3C-fwd	CAAGGGTTCACCGAAAGTTCACTGAGGTTCACCGAAAGTTCAAGTCGGTTCACCGAAAGTTCACTGC
bRARE-3C-rev	GGCCGCAGTGAACTTTCGGTGAACCGACTTGAACTTTCGGTGAACCTCAGTGAACTTTCGGTGAACCCTTGTAC
CMV-infus_left	TCTCGAGCTCAAGCTCTTCGCTATTACGCCAGTCG
CMV-infus_right	TCACCATGGTGGATCGACGTCCCCAGGCAGAAT
ef1a_Hind_left	GGTTAAGCTTGGAGAAGAGCATGCGTGAG
ef1a_Bam_right	GGTTGGATCCTCACGACACCTGAAATGGAA
venus_prom_seq_left	GGCCTTTTGCTCACATGTTC
VenusNLS_EcoRV_right	GCGTGATATCGGCCCAATGCATTGGC
