# Supplementary material for: The living microarray: a high-throughput platform for measuring transcription dynamics in single cells
Source: BMC Genomics. 2011 Feb 16;12:115. doi: 10.1186/1471-2164-12-115 (PMC3050818; doi:10.1186/1471-2164-12-115)

Supplementary Methods and Data (Plasmid Construction)

"The Living Microarray: a High-Throughput Platform for Measuring Transcription Dynamics in Single Cells". Rajan S, Djambazian H, Chu Pham Dang H, Sladek R and Hudson TJ

Plasmid Construction

See Additional file 1, Table S2 for the sequences for all primers used in plasmid construction.

$(GRE)_{x3}$-AdMLP-d2EGFP (Figure S8): An enhanced polylinker was created by subcloning an oligonucleotide insert (formed by annealing MCS_fwd and MCS_rev) into the KpnI/BamHI sites of pd2EGFP-1 (Clontech) to generate pd2EGFP-mod. Oligonucleotide cassettes containing the core AdMLP (AdMLP_fwd and AdMLP_rev) and a three-copy consensus GRE sequences (GRE-3C_fwd and GRE-3C_rev) were subcloned into the KpnI/PmeI and SalI/KpnI sites, respectively to generate $(GRE)_{x3}$-AdMLP-d2EGFP-mod. The promoter and coding sequences were then subcloned into pBluescript KS+ (Stratagene).

Venus-ECFP5 (Figure S9): This construct was cloned in three steps. First, the 1.2 kb promoter sequence of human EF1α was amplified by PCR from genomic DNA using primers Ef1a-1206_left and Ef1a-1206_right and subcloned into the AseI and AgeI (blunt) sites of pECFP-N1 (Clontech). The $(NLS)_{x3}$ cassette was amplified from CMV-dsRed-Nuc (Clontech) primers NLS-BsrG1-left and NLS-Not1-right and subcloned into the BsrGI/NotI sites to generate Ef1a-ECFP-NLS (Figure S7). Second, Venus-NLS/PEST-mod was generated by PCR amplification of the Venus-NLS/PEST sequence with primers VenusNLS-left and VenusNLS-Nhe-Asc-EcoRV-right from pCS2-Venus-NLS/PEST (21) and subcloning into the BamHI/HpaI sites of pd2EGFP-mod. The Ef1a-ECFP-NLS cassette was then amplified using primers Ef1a-nhe-left and Ef1a-asc-right and subcloned into the AscI/NheI sites of Venus-NLS/PEST-mod to generate Venus-ECFP4. Finally, to insulate the two fluorophore units, a synthetic polyadenylation sequence was amplified from pGL3-Basic (Promega) using primers pA-nhe-left and pA-nhe-right and subcloned into Venus-ECFP4 at the NheI site.

$(GRE)_{x3}$-AdMLP-VC5 (Figure S10): This construct was formed by subcloning the BamHI fragment from GRE-AdMLP-d2EGFP into the BamHI site of Venus-ECFP5.

(RARE)$_{x3}$-AdMLP-VC5 (Figure S10): This clone involved three steps. First, AdMLP-VC5 was formed by subcloning the AdMLP core promoter from AdMLP-d2EGFP into the KpnI/PmeI sites of Venus-ECFP5. Second, RARE-Venus-mod was formed by subcloning a synthetic oligonucleotide containing a three-copy consensus RARE sequence (by annealing bRARE-3C-fwd and bRARE-3C-fwd) into Venus-NLS/PEST-mod at the KpnI/NotI sites. The final plasmid was made by subcloning the SalI/NotI (blunt) digested RARE sequence into the SalI/KpnI (blunt) sites of AdMLP-VC5.

CMV-VC5 (Figure S1): This vector was generated by PCR-amplification of the CMV promoter from ECFP-N1 using primers CMV_infus_left and CMV_infus_right, and cloned into Venus-ECFP5 using the Infusion kit (Clontech).

EF1α-VC5 (Figure S1): This construct was cloned by PCR amplification of the EF1α promoter from EF1α-ECFP with primers ef1a_Hind_left and ef1a_Bam_right and subcloning it into the HindIII/BamHI sites of Venus-ECFP5.

TetRE-VC5 (Figure S1): This vector was formed by isolating the unidirectional Tetracycline-responsive promoter from pTRE-Tight-Bi (Clontech) at XhoI/KpnI sites and subcloning it into Venus-ECFP5 at SalI/KpnI sites.

(GRE)$_{x3}$-AdMLP-GV3 (Figure S2): This construct was cloned by amplifying the (GRE)$_{x3}$-AdMLP-Venus-NLS-PEST fragment from (GRE)$_{x3}$-AdMLP-VC5 using primers venus_prom_seq_left and VenusNLS_EcoRV_right and subcloning it into the HindIII/XbaI(blunt) digested pGL3 Basic (Promega).

Figure S7: Construction of EF1α-ECFP-NLS. The 1.2kb human EF1α promoter was cloned upstream of ECFP. We also fused the ECFP open reading frame to the SV40 nuclear localization signal to concentrate ECFP signal to the nucleus.

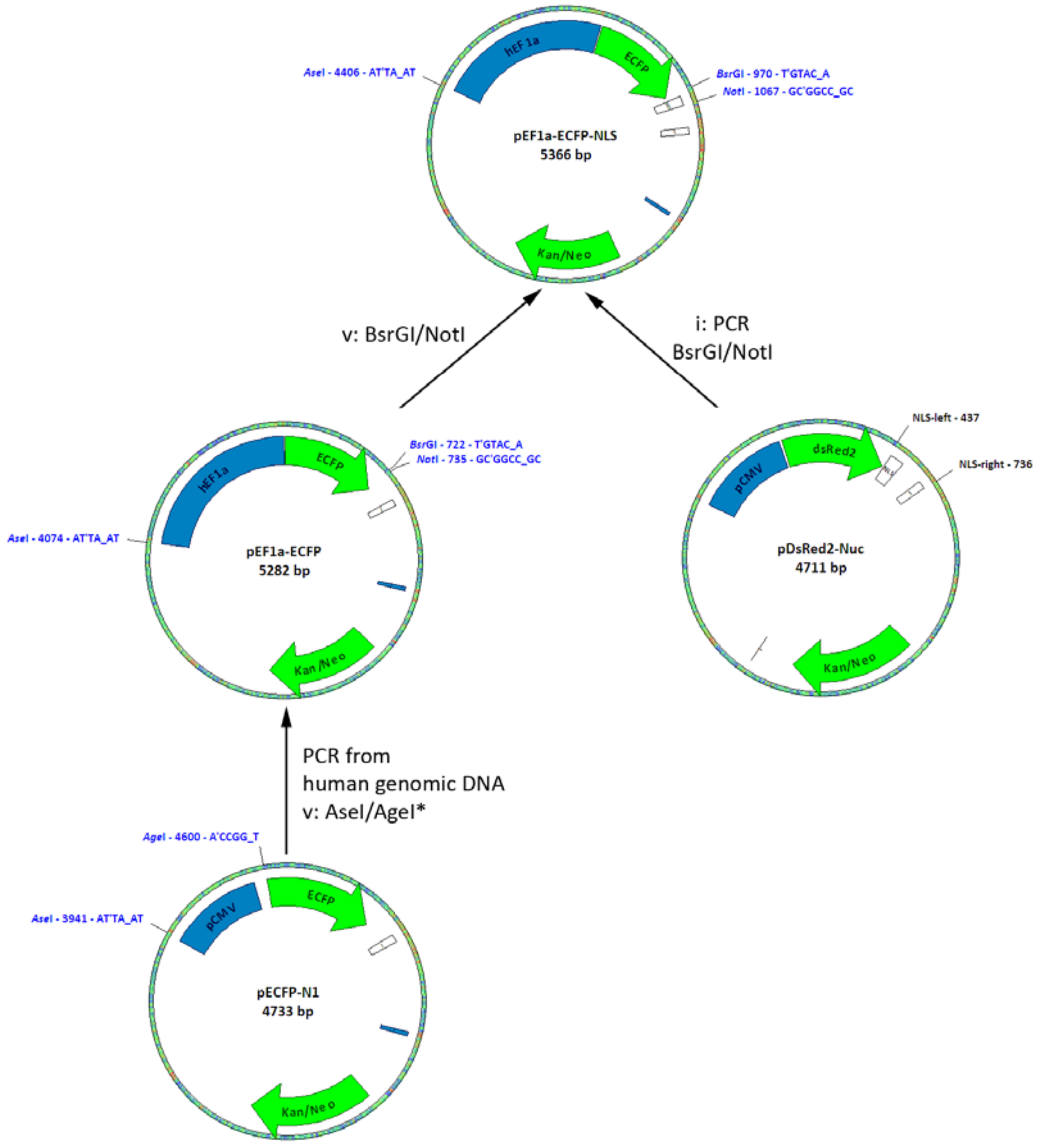

Figure S8: Construction of pGRE-AdMLP-d2EGFP. A three-copy consensus GRE was cloned upstream of the minimal AdMLP promoter into a modified polylinker for d2EGFP-1.

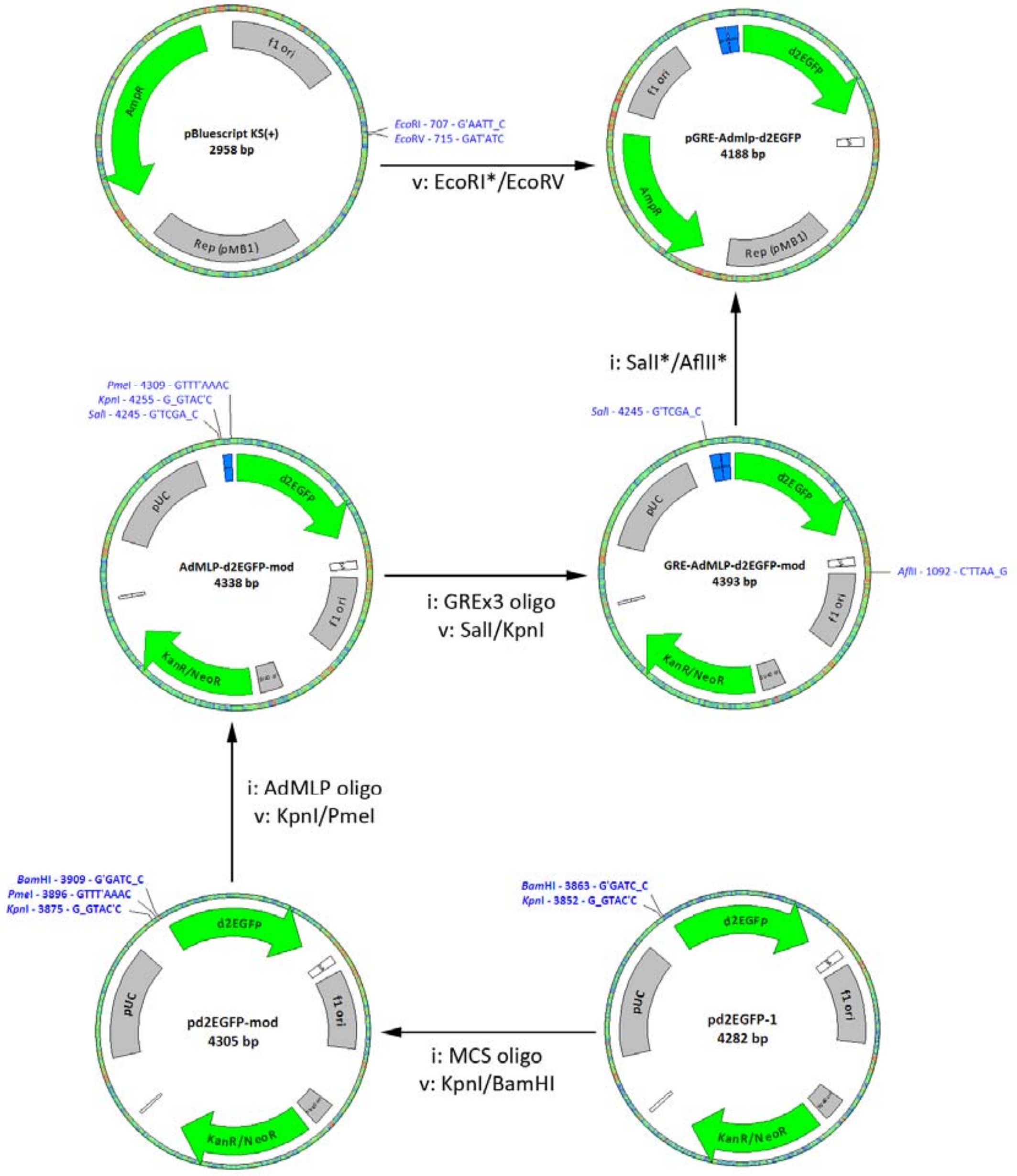

Figure S9: Construction of pVenus-ECFP5. This construct contains the Venus-NLS-PEST sequence downstream of an enhanced multiple cloning site. We also cloned the nuclear-localized, constitutively active ECFP cassette downstream of Venus and insulated the two genes with a poly adenylation signal.

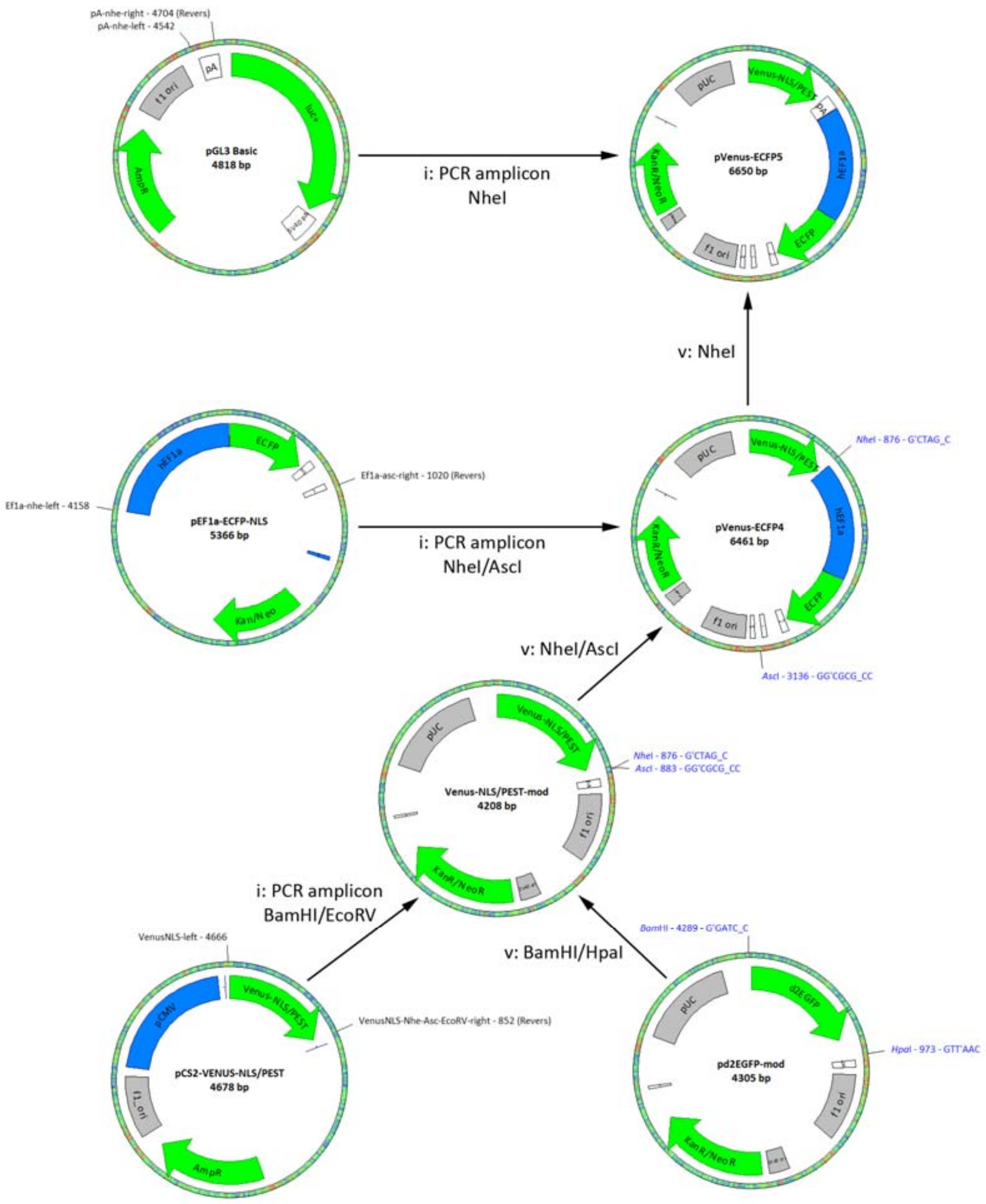

Figure S10: Construction of nuclear receptor based dual-fluorophore vectors. Three-copy consensus GRE and RARE elements were cloned into VC5 upstream of the AdMLP minimal promoter.

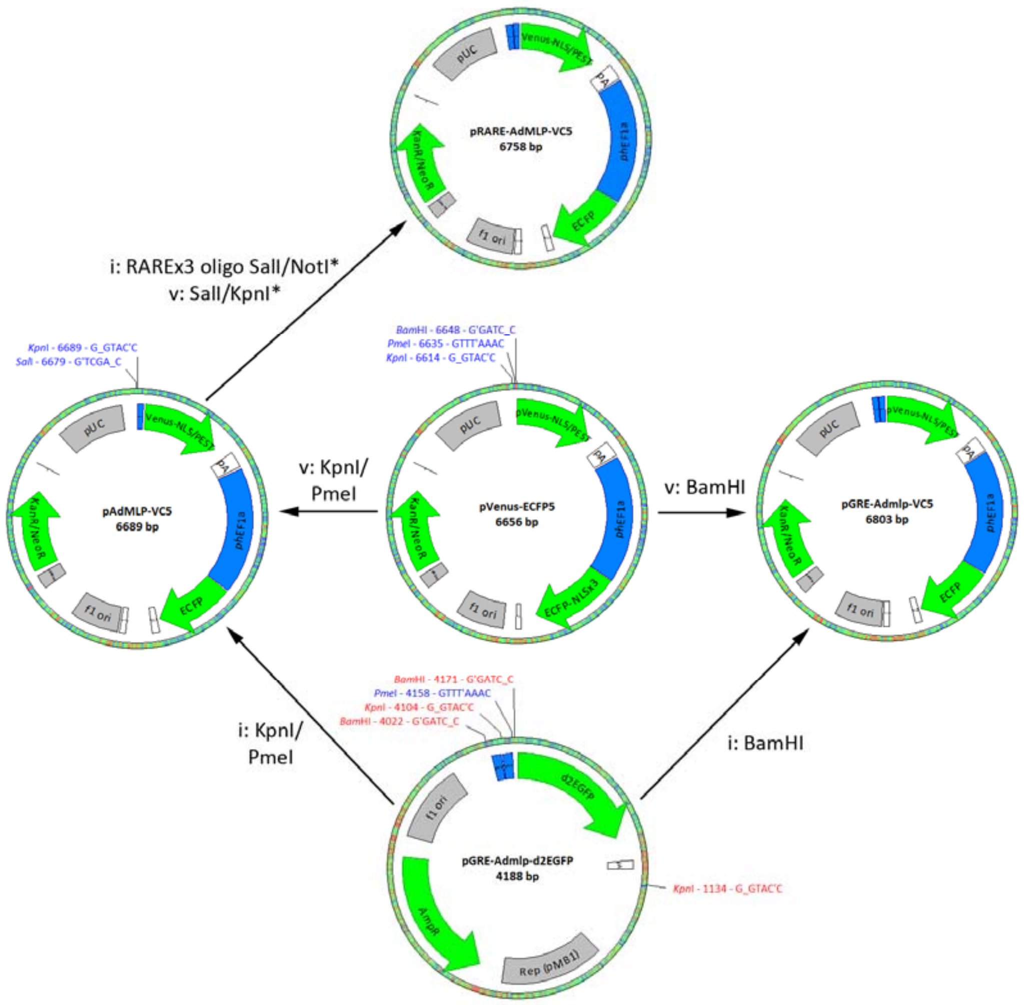

Figure S1: Construction of CMV, EF1α and TetRE based dual-fluorophore vectors. Two positive controls were made by cloning the EF1α and CMV promoters in VC5. An tetracycline-inducible VC5 construct was also made from the unidirectional promoter from pTRE-Tight-Bi of the Tet-On system.

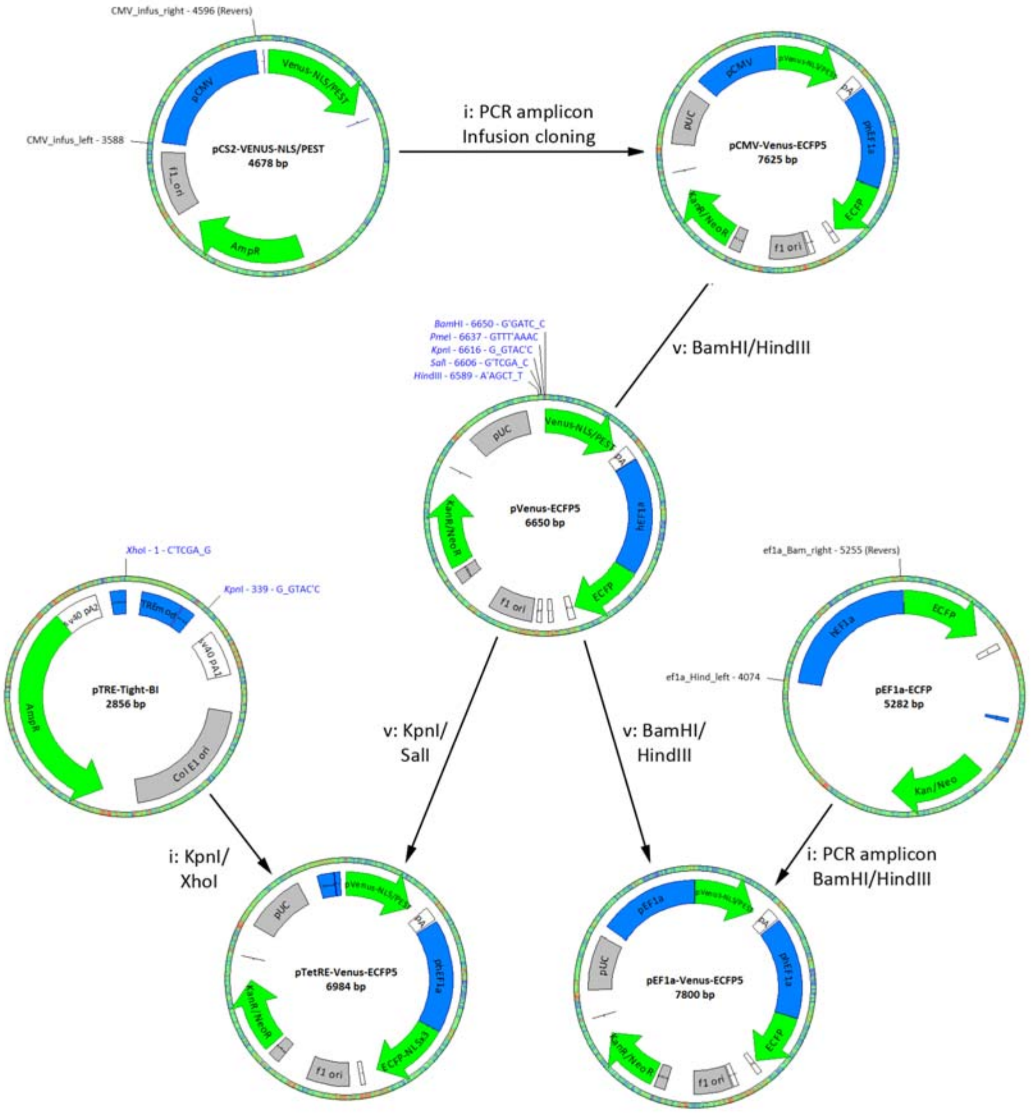

Figure S12: **Construction of (GRE)$_{x3}$-AdMLP-GV3.** This fluorescent reporter places Venus-NLS-PEST under the control of a three-copy consensus GRE and the AdMLP minimal promoter.

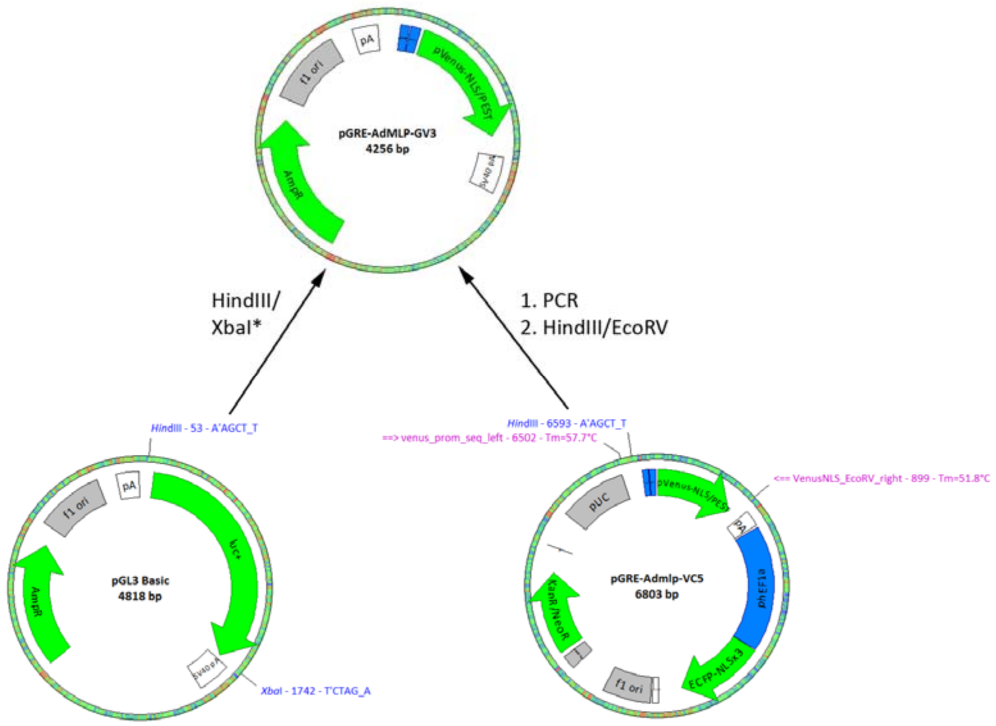

Supplement: Additional file 2 — Supplementary Methods and Data (Plasmid Construction). Contains methods used to construct the plasmids used in the manuscript, including vector maps. [file 1471-2164-12-115-S2.PDF]
